# Neocortical tissue recovery in severe congenital obstructive hydrocephalus after intraventricular administration of bone marrow-derived mesenchymal stem cells

**DOI:** 10.1186/s13287-020-01626-6

**Published:** 2020-03-17

**Authors:** María García-Bonilla, Betsaida Ojeda-Pérez, María L. García-Martín, M. Carmen Muñoz-Hernández, Javier Vitorica, Sebastián Jiménez, Manuel Cifuentes, Leonor Santos-Ruíz, Kirill Shumilov, Silvia Claros, Antonia Gutiérrez, Patricia Páez-González, Antonio J. Jiménez

**Affiliations:** 1 Departamento de Biología Celular, Genética y Fisiología, Universidad de Málaga, Campus de Teatinos, 29071 Malaga, Spain; 2 Instituto de Investigación Biomédica de Málaga (IBIMA), Malaga, Spain; 3 BIONAND, Andalusian Centre for Nanomedicine & Biotechnology (Junta de Andalucía-Universidad de Málaga), Malaga, Spain; 4 Department of Molecular Biology and Biochemistry, University of Seville, Seville, Spain; 5 Centro de Investigación Biomédica en Red sobre Enfermedades Neurodegenerativas (CIBERNED), Madrid, Spain

**Keywords:** Hydrocephalus, Bone marrow-derived mesenchymal stem cells, Spectroscopy, Reactive astrocytes

## Abstract

**Background:**

In obstructive congenital hydrocephalus, cerebrospinal fluid accumulation is associated with high intracranial pressure and the presence of periventricular edema, ischemia/hypoxia, damage of the white matter, and glial reactions in the neocortex. The viability and short time effects of a therapy based on bone marrow-derived mesenchymal stem cells (BM-MSC) have been evaluated in such pathological conditions in the hyh mouse model.

**Methods:**

BM-MSC obtained from mice expressing fluorescent mRFP1 protein were injected into the lateral ventricle of hydrocephalic hyh mice at the moment they present a very severe form of the disease. The effect of transplantation in the neocortex was compared with hydrocephalic hyh mice injected with the vehicle and non-hydrocephalic littermates. Neural cell populations and the possibility of transdifferentiation were analyzed. The possibility of a tissue recovering was investigated using ^1^H High-Resolution Magic Angle Spinning Nuclear Magnetic Resonance (^1^H HR-MAS NMR) spectroscopy, thus allowing the detection of metabolites/osmolytes related with hydrocephalus severity and outcome in the neocortex. An in vitro assay to simulate the periventricular astrocyte reaction conditions was performed using BM-MSC under high TNFα level condition. The secretome in the culture medium was analyzed in this assay.

**Results:**

Four days after transplantation, BM-MSC were found undifferentiated and scattered into the astrocyte reaction present in the damaged neocortex white matter. Tissue rejection to the integrated BM-MSC was not detected 4 days after transplantation. Hyh mice transplanted with BM-MSC showed a reduction in the apoptosis in the periventricular neocortex walls, suggesting a neuroprotector effect of the BM-MSC in these conditions. A decrease in the levels of metabolites/osmolytes in the neocortex, such as taurine and neuroexcytotoxic glutamate, also indicated a tissue recovering. Under high TNFα level condition in vitro, BM-MSC showed an upregulation of cytokine and protein secretion that may explain homing, immunomodulation, and vascular permeability, and therefore the tissue recovering.

**Conclusions:**

BM-MSC treatment in severe congenital hydrocephalus is viable and leads to the recovery of the severe neurodegenerative conditions in the neocortex. NMR spectroscopy allows to follow-up the effects of stem cell therapy in hydrocephalus.

## Background

In congenital hydrocephalus, there is an active accumulation of cerebrospinal fluid with a prevalence of 4–6 individuals per 10,000 births [1, 2]. Clinically, it manifests with ventriculomegaly (expansion of the cerebral ventricles) and increased intracranial pressure [3], thus adversely affecting brain tissue enclosed by the skull [4]. The main pathological consequences of congenital hydrocephalus include damage to the cerebral white matter, ischemia/hypoxia, inflammation, edema, and gliosis [4–6]. There is no cure for congenital hydrocephalus, and presently, non-surgical therapies are not compelling [7]. Thus, current treatments are surgical and include ventricular shunts, extraventricular drains, or third ventriculostomy. All of these treatments are merely palliative and prevent some, but not all the associated damages [4, 8]. Implantation of a ventricular shunt is the most common treatment but presents frequent complications such as obstruction, infection, fracture, migration, overdrainage, or underdrainage [9, 10]. For this reason, alternative strategies, such as stem cell therapies, have been proposed hopeful in the treatment of hydrocephalus [11].

Mesenchymal stem cells (MSC) are considered an efficient source for cell-based therapies in neurodegenerative diseases. MSC can be purified from the bone marrow stromal population based on the criteria that has been indicated by the International Society for Cellular Therapy [12]. For this study, following the criteria of isolation, a heterogeneous non-clonal culture of stromal cells that contains stem cells with different multipotential properties committed progenitors and differentiated cells has been obtained [12]. Therefore, in this study, the term MSC will actually consider the stem cells referred to those conditions of isolation. MSC expand efficiently in vitro, retain their multipotent potential, and generate high cell quantities [13]. In experimental therapies for chronic neurodegenerative diseases, MSC provide functional improvements [14], which has been attributed to their production of neurotrophins, reduction of oxidative stress, and modulation of inflammatory responses [13, 14]. MSC also play a neuroprotective role by creating favorable environments for regeneration producing growth factors and cytokines and promoting vascularization and remyelination [15].

Severe obstructive hydrocephalus concurs with the physical deterioration of the neocortical white matter by periventricular edema and astrogliosis [4, 16, 17]. Clinical consequences at this stage include frequent shunt failures that are difficult to treat [18]. We therefore propose that intracerebroventricular administration of bone marrow-derived MSC (BM-MSC) could provide clinical benefits beyond the current standard of care.

The etiology of hydrocephalus that occurs in the hyh mouse [19, 20] is similar to diverse forms of human fetal-neonatal hydrocephalus [21–23]. This animal model is compatible and comparable to hydrocephalic infants undergoing complications [20, 24], exhibiting similar neuropathological events such as myelin damage and glial reactions [20].

Neuropathologically, in the hyh mouse, an obstruction of the cerebral aqueduct [25] induces severe hydrocephalus by the post-natal first week and mortality within a few more weeks [26]. The ventricular obstruction is motivated by an alteration affecting the development of the neuroepithelium covering the brain ventricles that lead to ependymal lost and ventricular obstruction [27, 28]. Neuroepithelial developmental failure is a consequence of the mutation in the *Napa* gene. This gene codifies the *N*-ethylmaleimide-sensitive factor attachment protein alpha (αSNAP) implicated in vesicular trafficking [27, 29].

Metabolite/osmolyte signatures have been previously defined for various stages in the pathology of hydrocephalus [30] and serve as appropriate biomarkers in the progression of the disease.

The present research has evaluated the viability and the therapeutic effect of BM-MSC therapy in severe congenital hydrocephalus by measuring metabolites/osmolytes levels that are present in the neocortical parenchyma. For this purpose, ^1^H High-Resolution Magic Angle Spinning Nuclear Magnetic Resonance (^1^H HR-MAS NMR) spectroscopy [30] has been used.

Results have shown that 4 days following BM-MSC intraventricular administration in hyh mice undergoing very severe hydrocephalus, undifferentiated BM-MSC are stabilized and mixed into the reactive astrocytes in the neocortex. A recovering in the neocortical tissue of hyh mice treated with BM-MSC was detected according to the levels of metabolites/osmolytes and the reduction of periventricular apoptosis. Due to the fact that most of the hyh mice with severe hydrocephalus are not able to survive more than 5 weeks [26], the treatment was evaluated 4 days after transplantation in 20-day-old mice. Thus, searching effects of longer treatments was not viable.

We can conclude that BM-MSC treatment is feasible and beneficial for severe hydrocephalus. Besides, spectroscopy could be transferable to clinical settings for evaluating stem cell therapies in hydrocephalus.

## Methods

### Experimental animals

Transgenic homozygous male and female mice expressing the monomeric red fluorescent protein 1 (mRPF1; Tg (GAG-mRPF1)aF1Hadj/J), of both sexes and 25 days of age, were used to obtain the BM-MSC. Mutant hyh mice (hydrocephalus with hop gait, B6C3Fe- a/a-hyh/J strain), 20-day-old males and females with very severe hydrocephalus according to Bátiz et al. [26], were used to transplant BM-MSC (hydrocephalic hyh BM-MSC-injected group) or sterile saline serum (hydrocephalic hyh sham-injected group). Non-hydrocephalic littermates at the same age were also used in the analysis of some parameters (non-hydrocephalic control littermates group, nh). Hyh and non-hydrocephalic mice were identified by phenotype inspection and genotyping [31]. All mice were originally obtained from The Jackson Laboratory (Bar Harbor, ME, USA) and bred by the Animal Experimentation Service of the University of Malaga, in a room at 22 °C with a 12:12 light/dark cycle, and standard food and water available ad libitum. The design of the experiments, housing, handling, care, and processing of the animals were conducted in accordance with European and Spanish laws (RD53/2013 and 2010/63UE), and following ARRIVE guidelines. According to current legislation, experimental procedures (protocol # 4-2015-A) were approved by the Institutional Animal Care and Use Committee of the University of Malaga (CEUMA, Spain) and the Regional Government Council (Junta de Andalucía, Spain).

### Bone marrow-derived mesenchymal stem cell isolation and culture

Transgenic mRPF1 mice were sacrificed by cervical dislocation to obtain the BM-MSC. Femurs were dissected out, and bone marrow was flushed with a 29-gauge needle syringe (320924, BD Microfine, Madrid, Spain) containing Dulbecco’s modified Eagle’s medium (DMEM, Sigma-Aldrich, St Louis, MO, USA) supplemented with 1% penicillin/streptomycin, 0.5% amphotericin B, 6.25% l-glutamine, and 10% fetal bovine serum (FBS, Sigma-Aldrich). The suspension was centrifuged at 400*g* for 5 min. The cell pellet was suspended in 14 ml of supplemented DMEM, plated on 75 cm^2^ flasks, and incubated in a humidified incubator at 37 °C with 5% CO_2_. Twice per week, the media were changed, and non-adherent hematopoietic cells were removed. After 7–10 days, when the culture was approximately 80% confluent, cells were detached with trypsin/ethylenediaminetetraacetic acid (EDTA; Sigma-Aldrich) and placed in new flasks. After confluence, BM-MSC were detached and centrifuged and resuspended in saline serum at 10,000 cells/μl. In some experiments, red fluorescent BM-MSC were also labeled with a green cell tracker dye (C2925, Molecular Probes, Thermo Fisher, Waltham, MA, USA) before their transplantation. For that purpose, flasks were rinsed with PBS and incubated with DMEM without FBS for 30 min followed by incubation in green cell tracker (1 μg/ml) for 30 min. There are variables that could potentially affect the efficiency of treatment with MSC. Thus, acute and chronic senescence has been reported affecting BM-MSC under some culture conditions [32]. These conditions were avoided in the experiments carried out in the present study. BM-MSC were always obtained from young transgenic mice. BM-MSC were always cultured under the same conditions and transplanted at passage 1 after reaching 70–80% confluence. Cells were not cryopreserved and used immediately. Immunogenicity, which is also relevant in transplants with BM-MSC [33], was avoided as possible, using allogenic transplants. Each experimental set was performed using a transgenic mouse as a donor to a few hydrocephalic hyh from the same litter or consanguineous breeders. The same researcher performed the BM-MSC isolations.

### Scanning electron microscopy analysis

For scanning electron microscopy, BM-MSC were placed in 13-mm-diameter non-coated coverslips. When BM-MSC were approximately 80% confluent, they were rinsed with phosphate buffer 0.1 M, pH 7.2 (PB), fixed with 2% buffered glutaraldehyde (Electron Microscopy Sciences, Hatfield, PA, USA), dehydrated in graded alcohol solutions, dried, and sputter-coated with gold.

### Flow cytometry analysis

BM-MSC from transgenic mRPF1 mice were detached with trypsin/EDTA from flasks and suspended in ice-cold Leibovitz’s L-15 medium (21083, Gibco, streptomycin/streptomycin, 200 mg of bovine serum albumin (BSA, A2058, Sigma-Aldrich), 1% HEPES, and 10% distilled H_2_O. BM-MSC suspensions (10^6^ cells/ml) were incubated for 30 min at 4 °C with fluorescent monoclonal antibodies (see Table 1) in the supplemented L-15 medium. To detect intracellular antigens, BM-MSC were detached as described above and fixed in 4% buffered paraformaldehyde for 10 min. Then, fixed BM-MSC were centrifuged and incubated with primary and fluorescent secondary antibodies as described below. For the analysis of DNA content, fixed BM-MSC were incubated with the nuclear dye 4′,6-diamidino-2-phenylindole (DAPI; Molecular Probes, Eugene, OR, USA; 5 μg/ml). A DakoCytomation MoFlo cytometer (Dako, Santa Clara, CA, USA) and an FD FACSverse system (BD Biosciences, Franklin Lakes, NJ, USA), calibrated according to the manufacturer’s recommendations, were used for the analysis. Fluorescence compensation was set using single-stained controls, and matching median compensation algorithms were applied. Data were analyzed using Summit 4.3.2. Software (Dako) and Kaluza Analysis Software (Beckman Coulter, Indianapolis, IN, USA). Strategies involving gating size versus granularity (forward scatter versus side scatter) and doublet exclusion were performed.

**Table 1:** Primary antibodies

Antibody	Source	Reference	Type	Use	Dilution
Aquaporin-4	Sigma-Aldrich	A5971	RbP	FR	1:200
BDNF	Abcam	ab108319	RbM	I, VB	1:250
CD11b	BioLegend	101225	RtM	FC	1:80
CD34	BioLegend	119309	MM	FC	1:25
CD44	BioLegend	103015	MM	FC	1:50
CD45.1	BioLegend	110713	MM	FC	1:80
CD73	BD	561543	RtM	FC	1:25
CD90.2	BioLegend	105315	RtM	FC	1:200
Collagen type II	Calbiochem-Novabiochem	AB746P	RbP	C	1:40
F4/80	Biolegend	123107	RtM	I, FC	1:200
GAPDH	Abcam	Ab9485	RbP	WB	1:2500
GDNF	Santa Cruz	sc-328	RbP	I, FC, VB	1:100
GFAP	Sigma	G-A-5	MM	I, FC, VB, WB	1:1000
δGFAP	Merck	AB9598	RbP	I	1:500
Glutamine synthetase	Abcam	Ab49873	RbP	VB	1:5000
Iba1	Wako	019-19741	RbP	FR, VB, WB	1:500, 1:1000 (WB)
Myeloperoxidase	Abcam	Ab139748	RbP	VB	1:100
Nestin	BIO-RAD	AHP1739	RbP	I, FC, VB	1:100
NeuN	Merck	MAB377	MM	I, FC, VB	1:100
NG2	Merck	AB5320	RbP	I	1:200
NGF	Abcam	AB6199	RbP	I, VB	1:500
RFP	Chromotek	5F8	RtM	FC, VB	1:500
β-III tubulin	Promega	A6712	MM	I, FC	1:5000
VEGF	Abcam	Ab46154	RbP	I, FC, VB	1:1000

*Abbreviations*: *C* BM-MSC in cell cultures, *I* immunofluorescence for BM-MSC on slides, *FC* flow citometry, *FR* frozen sections, *MM* mouse monoclonal, *RbM* rabbit monoclonal, *RtM* rat monoclonal, *RP* rabbit polyclonal, *VB* vibratome sections, *WB* western blot. Sources: AbCam, Cambridge, UK; BD, San Diego, CA, USA; BioLegend, San Diego, CA, USA; BIO-RAD, Oxford, UK; Chromotek, Planegg-Martinsried, Germany; GeneTex, Irvine, CA, USA; Merck Millipore, Burlington, MA, USA; Promega, Madison, WI, USA; Santa Cruz Biotechnology, Dallas, TX, USA; Sigma-Aldrich

### In vitro multilineage cell differentiation

Trilineage differentiation capacity was assessed for BM-MSC. For adipogenic differentiation, BM-MSC were cultured at a density of 3 × 10^5^ cells/cm^2^ with DMEM 10% FBS. Once confluence was reached, the culture medium was replaced with adipogenic induction medium: DMEM containing 10% FBS, 15% rabbit serum (16120107, Gibco), 10^−7^ M dexamethasone, 0.5 mM 3-isobutyl-1-methylxanthine, 10^−9^ M bovine insulin (I6634, Sigma-Aldrich), and 0.2 M indometacin. In cells fixed with 4% paraformaldehyde, on days 4, 7, and 21, Oil Red O staining was used to detect lipid vacuoles (indicative of adipogenic differentiation). Chondrogenic differentiation was assayed using suspensions of 10^6^ cell/ml to form 3D pellets in conical tubes with DMEM supplemented with 10^−7^ M dexamethasone, 1% ascorbate-2-phosphate, 1% insulin-transferrin-selenium (ITS) + Premix Tissue Culture Supplement (354352, Corning, New York City, NY, USA), 1 mM sodium pyruvate, 1% MEM non-essential amino acid solution (Sigma, M7145), and 10 ng/ml recombinant human transforming growth factor-beta 1 (rhTGF-β1, 240-B, R&DSystems, Minneapolis, MN, USA). Pellet culture without rhTGF-β1 was used as negative control. After 21 days, pellets were fixed with 10% of neutral buffered formalin. Then, they were dehydrated in alcohol, embedded in paraffin, and sectioned at 10 μm thick. To detect cartilage matrix, indicating chondrogenic differentiation, collagen type II was immunodetected, after hydration of the paraffin sections, using an appropriate antibody (Table 1) followed of a biotinylated secondary antibody, ExtrAvidin-peroxidase (diluted 1:2000; E2886, Sigma), and 3,3′-diaminobenzidine tetrahydrochloride (DAB; Sigma; 0.1%) as chromogen. Then, sections were dehydrated and mounted. Sections were also stained with Toluidin Blue, Blue Alcian, and Safranin O-Fast Green to check the metachromatic reaction of the cartilage matrix. For osteogenic differentiation assay, BM-MSC were cultured at a 3 × 10^3^ cells/cm^2^ density with DMEM + 10% FBS. When cultures were approximately 80% confluent, the medium was supplemented with 10^−8^ M dexamethasone, 50 μM ascorbate-2-phosphate, and 2 mM β-glycerophosphate. Alkaline phosphatase (ALP) histochemistry/fluorometry (for details of the procedures, see [34]) and Alizarin Red S staining were used to detect calcium deposition on days 7, 14, and 21 after induction. In the adipogenic and osteogenic differentiation assays, BM-MSC with only DMEM + 10% FBS were cultured for negative control. For the chondrogenic assay, pellet culture without rhTGF-β1 was used as the negative control.

### Bone marrow-derived mesenchymal stem cell characterization

BM-MSC were detached with trypsin/EDTA, centrifuged at 400*g* for 5 min, and fixed in 2% buffered paraformaldehyde for 10 min. Then, BM-MSC were placed on slides, dried at room temperature, and processed for immunofluorescence as described below. More than 7000 BM-MSC were analyzed in these conditions.

### Bone marrow-derived mesenchymal stem cell transplantation

Procedures were performed under 3% sevoflurane in 1 l/min of oxygen anesthesia in an adapted stereotaxic instrument. A hole (1 mm diameter) was drilled in the skull covering the distinguishable enlarged right lateral ventricle of hydrocephalic hyh mice. Then, a 20-μl cell suspension containing 200,000 BM-MSC was injected into the ventricle with a peristaltic pump at 10 μl/min. The needle was left 2 min more before removing, and finally, the skin was closed. A group of hyh hydrocephalic mice (hydrocephalic hyh sham-injected) were injected following the same procedure with vehicle solution (saline) but no cells.

### ^1^H HR-MAS NMR spectroscopy

Non-hydrocephalic mice (*n* = 20), hydrocephalic hyh mice transplanted with BM-MSC (*n* = 10), and hydrocephalic hyh sham-injected mice (*n* = 9) were sacrificed by cervical dislocation. The neocortex of the right and left hemispheres was quickly removed under cold conditions, frozen on dry ice, and stored at − 80 °C. ^1^H HR-MAS NMR spectroscopy was used to analyze the metabolic profiles of dissected samples from the right hemispheres following the previously described methodology [30]. Spectra were obtained in a 600-MHz Bruker Avance Spectrometer (Bruker BioSpin, Ettlingen, Germany), at 4 °C and a 5-kHz spinning rate.

### RT-PCR

The neocortex of the left hemispheres from the same mice used for ^1^H HR-MAS NMR spectroscopy and non-injected hydrocephalic hyh mice (without any surgery, *n* = 7) was used to extract total RNA with Tripure Isolation Reagent (Roche, Basel, Switzerland). Contaminating DNA was removed using DNAase (Sigma-Aldrich). Retrotranscription of 3 μg of total RNA was performed with the High-Capacity cDNA Reverse transcription Kit (4374967, Applied Biosystems, Foster City, CA). For RT-PCR, 40 ng of cDNA was mixed with Eagle Taq Master Mix (Sigma-Aldrich) and TaqMan Gene Expression assay probes (IL-1α Mm01336161_m1, IL-1β Mm00434228_m1, IL-10 Mm00439614_m1, CD45 Mm01293577_m1, GFAP Mm01253033_m1, iba1 Mm00479862_gl, and Ki67 Mm01278617_m1; Applied Biosystems). Quantification was performed with an ABI Prism 7000 Sequence Detector System (Applied Biosystems). Results were expressed using the comparative double-delta Ct method (2-ΔΔCt). ΔCt values represent glyceraldehyde-3-phosphate dehydrogenase (GAPDH) normalized expression levels and non-hydrocephalic mice as a control condition. In all cases, the slopes of the curves indicated optimal PCR conditions (slopes of 3.2–3.4).

### Western blot analysis

The neocortex from the same mice used for metabolite and RT-PCR analysis was also used for the western blot analysis. Total protein was extracted using RIPA buffer with a protease inhibitor cocktail (P8340, Sigma-Aldrich) for 10 min at 4 °C. Then, samples were centrifuged at 8000*g* for 10 min at 4 °C. Proteins from the supernatant were quantified with the Bradford assay and separated on a one-dimensional SDS-polyacrylamide gel electrophoresis, transferred to a 0.45-μm-pore-diameter nitrocellulose membrane Amersham PROTAN (Sigma-Aldrich) in a SemiDry TRANS-BLOT (BIO-RAD, Hercules, CA, USA), incubated with the primary antibodies (see Table 1), and then in HRP-conjugated secondary antibodies (Sigma-Aldrich). Immunoreaction was visualized with a chemiluminescent substrate (ECL, Cell Signalling, Danvers, MA, USA) and imaged using ChemiDoc XRS+ (BIO-RAD). GAPDH was used as a protein loading control and non-hydrocephalic mice as a control condition.

### Immunofluorescence

Mice (hydrocephalic sham-injected mice, *n* = 11; hydrocephalic hyh mice transplanted with BM-MSC, *n* = 12; non-hydrocephalic mice, *n* = 5) were sacrificed under anesthesia with Dolethal (sodium pentobarbital; Vétoquinol, Lure, France; intraperitoneal administration, 0.2 mg/g body weight) and transcardially perfused with 4% buffered paraformaldehyde. Fixed brains were removed and post-fixed in the same solution for 24 h at 4 °C. The brains were sectioned with a vibratome (80-μm-thick sections) or were cryoprotected in 30% sucrose to obtain frozen sections (60-μm-thick). Type of sections and thickness were selected accordingly to obtain the best immunolabeling depending on the used antibodies and to maintain the section integrity. Sections of brains and slides containing isolated BM-MSC were immunostained with specific primary antibodies and appropriate fluorescent secondary antibodies (Table 1), using DAPI as nuclear staining, and mounted in Vectashield (Dako, Glostrup, Denmark).

### Apoptosis analysis

Additional groups of experimental mice (hydrocephalic hyh sham-injected mice, *n* = 6; hydrocephalic hyh mice transplanted with BM-MSC, *n* = 5) were sacrificed and fixed as described above for immunofluorescence. Brains were cryoprotected in 30% sucrose to obtain 60-μm-thick frozen sections. Apoptotic cells in the neocortex were detected using a TUNEL assay (ab206386, Abcam, Cambridge, UK) according to the manufacturer’s instructions.

### In vitro assay of bone marrow-derived mesenchymal stem cells under TNFα conditions

BM-MSC in P1 (70–80% confluence) were plated in DMEM (supplemented with 1% penicillin/streptomycin, 0.5% amphotericin B, and 6.25% l-glutamine) containing 50 ng/ml TNFα (cyt-252-a; Prospec, Ness-Ziona, Israel) and incubated for 24 h with 5% CO_2_ at 37 °C. Two control conditions were used: (i) C1, incubated in DMEM with no TNFα and supplemented with 1% penicillin/streptomycin, 0.5% amphotericin B, and 6.25% L-glutamine; (ii) C2, incubated in same culture medium used for C1 but supplemented with additional 10% FBS. After incubation, media from the different conditions were frozen at − 80 °C and stored until cytokine and mass spectrometry analyses were performed.

### Cytokine analysis of culture media

Culture media from BM-MSC with TNFα and the controls C1 and C2 were incubated on membranes for a mouse 62 cytokines antibody array (Abcam, AB133995) and processed according to the manufacturer’s instructions. Densitometry data was calculated using Image Lab software (BIO-RAD) and normalized in base to C1 control. A heat map for cytokine expression (densitometry) was obtained using http://heatmapper.ca [35]. The PANTHER Classification System (v.14.1) [36] and DAVID Bioinformatic Resources (6.8) [37] were used to detect the main biological processes related to the overexpressed proteins.

### Mass spectrometry analysis of the secretome in the culture media

Peptide analysis of the culture media samples from BM-MSC under TNFα and C1 conditions (3 samples each condition) was carried out by quadrupole-orbitrap nano-HPLC-ESI-MS/MS. Culture media was lyophilized and dissolved in water. Gel-assisted proteolysis was performed for the proteins entrapped in a polyacrylamide gel matrix. Protein digestion and sample preparation were carried out using DigestPro MSI (INTAVIS Bioanalytical Instruments AG, Cologne, Germany). Peptides were purified and concentrated using C18 ZipTip (Merck Millipore) according to the manufacturer’s instructions. Samples were injected into an Easy nLC 1200 UHPLC system coupled to a Q Exactive™ HF-X Hybrid Quadrupole-Orbitrap Mass Spectrometer (ThermoFisher). Data was acquired using Tune 2.9 and Xcalibur 4.1.31.9 (ThermoFisher). Swiss-Prot database was used to identify *Mus musculus* proteins. These proteins were analyzed using Proteome Discoverer 2.2 (Thermo Fisher) and the tandem mass spectrometry data analysis program SEQUEST. The false discovery rate (FDR) was calculated using Percolator. Minora feature detector in Proteome Discoverer 2.2 (Thermo Fisher) was used for the label-free quantification.

### Image analysis and quantification

Immunofluorescence images were obtained with Leica SP5 II and SP8 laser confocal microscopes (Leica, Wetzlar, Germany). For each experiment, images were obtained in batches using the same settings. Figures were composed using Adobe Photoshop CS5.1, and the same minimal changes in brightness and contrast were applied. The densities (cells/area) of GFAP+, Iba1+, NG2+, Olig2+, and NeuN+ cells were calculated in 4 fields in each parietal cortex section (2-μm-thick confocal planes) per animal. Western blot images obtained with ChemiDoc XRS+ were quantified using ImageJ software (NIH, USA). Densities of apoptotic cells were quantified in bright-field micrographs obtained by scanning under a VS120 microscope (Olympus, Tokyo, Japan) with an UPLSAPO20x/0.75 objective, and then processed with ImageJ software. The whole periventricular areas of 3 sections per animal (60-μm-thick sections) were used for quantification of TUNEL+ nuclei.

### Statistics

Statistical analyses were performed using KaleidaGraph (Synergy Software, Reading, PA, USA) and Statgraphics Centurion XVII (Statpoint Technologies, Warrenton, VA, USA). The required sample size was estimated according to standard deviations or significances with a minimum of 30 freedom degrees. Animals were numbered without indication of the group. Data collection and analyses were blinded by using different researchers and by masking the samples. All values are reported in the figures as mean with 95% confidence. The Wilcoxon-Mann-Whitney test and Student’s *t* test were applied for hypothesis testing in situations requiring non-parametric and parametric analyses, respectively. When the *F* probability from Student’s *t* test was < 0.05, the variance was considered unequal. *P* < 0.05 based on both tests was considered statistically significant.

A logistic regression analysis was carried out to identify the differential metabolite profiles that distinguish both groups of hydrocephalic hyh mice (treated with BM-MSC and sham-injected). A prediction exponential function was obtained. The relationship between the variables *X*_*n*_ and *P* is non-linear, and the parameters *B*_*n*_ were estimated using the logistic regression algorithm. Larger *B*_*n*_ values indicated a larger impact of the variables *X*_*n*_.

$$ P\ \left[v=2\right]=\exp \left({B}_0+{B}_1{X}_1+\dots +{B}_n{X}_n\right)/1+\exp \left({B}_0+{B}_1{X}_1+\dots +{B}_n{X}_n\right) $$

$$ Z={B}_0+{B}_1{X}_1+\dots +{B}_n{X}_n $$

*P* is the predicted probability of a given sample to be classified in 1 of the 2 defined groups (BM-MSC and sham). *V* is the variable of the group and indicates 1 of the 2 groups analyzed.

In PANTHER and mass spectrometry analyses, *P* value calculation is indicated in the tables.

## Results

### Multipotentiality and characterization of the BM-MSC

**Fig. 1:**
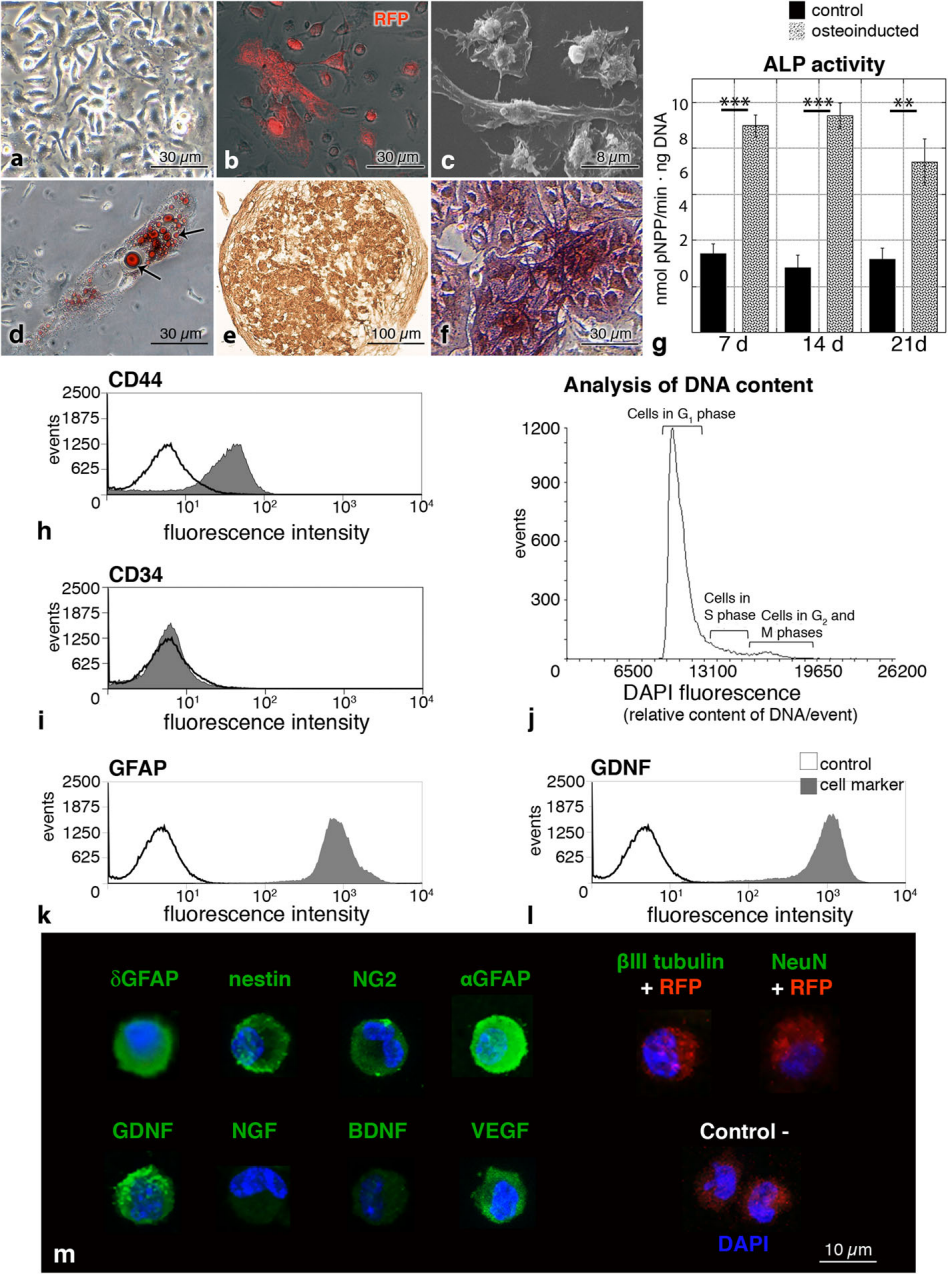
Characterization of BM-MSC before transplantation into hydrocephalic hyh mice. **a** BM-MSC primary culture forming a colony unit. Phase-contrast microscopy. **b** BM-MSC expressing the mRFP1 (red). Merge of phase-contrast and epifluorescence microscopies. **c** Spindle-shaped BM-MSC under scanning electron microscopy. **d** Oil Red O staining of lipid droplets (arrows) in the cytoplasm of BM-MSC after adipogenic differentiation for 14 days. Merge of phase-contrast and bright-field microscopy images. **e** Collagen type II immunostaining of a 3D pellet culture of BM-MSC after 21 days of chondrogenic differentiation. **f** Osteoinducted BM-MSC stained with alkaline phosphatase (ALP) 21 days after induction. **g** ALP activity in control (black) and osteoinducted (gray) BM-MSC at 7, 14, and 21 days. **h**, **i** Representative immunophenotype profiles of unfixed BM-MSC for CD44 and CD34 markers by flow cytometry. **j** Flow cytometry analysis of DNA content (DAPI fluorescence) in fixed BM-MSC. **k**, **l** Detection of a neural cell marker (GFAP) and a neuroprotector factor (GDNF) in fixed BM-MSC by flow cytometry. **m** Immunofluorescence (green) in fixed BM-MSC before injection for δGFAP, nestin, NG2, αGFAP, β-III tubulin, NeuN, GDNF, NGF, BDNF, and VEGF. In the absence of labeling, the channel for red (RFP fluorescence) is also shown. Nuclear staining with DAPI (blue). Negative control represents the omission of the primary antibodies. **P < 0.02, ***P < 0.01 Wilcoxon-Mann-Whitney test

BM-MSC from mRFP1 transgenic mice were grown in primary cultures until forming a large number of colony-forming units (Fig. 1a). On secondary cultures, BM-MSC retained their red fluorescence and appeared as spindle-shaped cells (Fig. 1b, c).

In order to confirm the identity of the isolated BM-MSC as multipotential cells [38], their trilineage differentiation was assayed through adipocytes, chondrocytes, and osteocytes. Adipogenic differentiation was confirmed by the presence of lipid droplets after 14 days with adipogenic-inducing culture medium (Fig. 1d). The 3D pellet culture showed that BM-MSC were able to differentiate into chondrocytes, exhibiting a cartilaginous matrix with collagen type II (Fig. 1e) and metachromasia. After osteogenic induction for 21 days, BM-MSC formed white nodule-like aggregations. Their mineralization was stained with alkaline phosphatase (ALP, Fig. 1f) and Alizarin Red. In osteoinducted cells, ALP activity was higher when compared to control cells (Fig. 1g). As a control, in the three lineages, non-supplemented cells were negative for all staining.

Flow cytometry analysis showed that non-fixed BM-MSC were positive for the mesenchymal markers [38] CD44, CD73, and CD90 (Fig. 1h, Additional file 1), and negative for the expression of the hematopoietic markers [38] CD34 and CD45 (Fig. 1i, Additional file 1). The analysis of the primary cell culture revealed that approximately 10% of the cells were positive for CD11b and F4/80 markers (Additional file 2). These profiles can be explained based on the analysis in the culture conditions and the cell passage (passage 1 in this case) [39]. The DNA content of BM-MSC was stained with DAPI and analyzed with flow cytometry (Fig. 1j). Proportions of cells in the different cycle phases were 89.2% in G_1_ (unreplicated complement of DNA), 4.3% in G_2_ and M phase (fully replicated complement of DNA), and 6.5% in S phase (intermediate amount of DNA). The presence of polyploid cells was discarded with DAPI staining in flow cytometry.

To characterize BM-MSC before their application in the hydrocephalic hyh mice, analyses of fixed cells by flow cytometry and immunofluorescence in slides were carried out (Fig. 1k–m). BM-MSC presented an eccentric kidney-shaped nucleus. BM-MSC were found expressing some neural stem cell markers, such as the glial fibrillary acidic protein delta (δGFAP), nestin, or neuron/glial antigen 2 (NG2). BM-MSC also expressed αGFAP (astrocyte marker). These markers were present in almost all the analyzed cells. On the other hand, only a few BM-MSC were labeled for β-III tubulin (neuroblast marker), and none for NeuN (mature neuron marker; Fig. 1m). Besides, BM-MSC were confirmed expressing neuroprotector factors (Fig. 1m), such as the glial cell-derived neurotrophic factor (GDNF), the neural growth factor (NGF), the brain-derived neurotrophic factor (BDNF), and the vascular endothelial growth factor (VEGF).

### Homing of the BM-MSC into the damaged periventricular walls

**Fig. 2:**
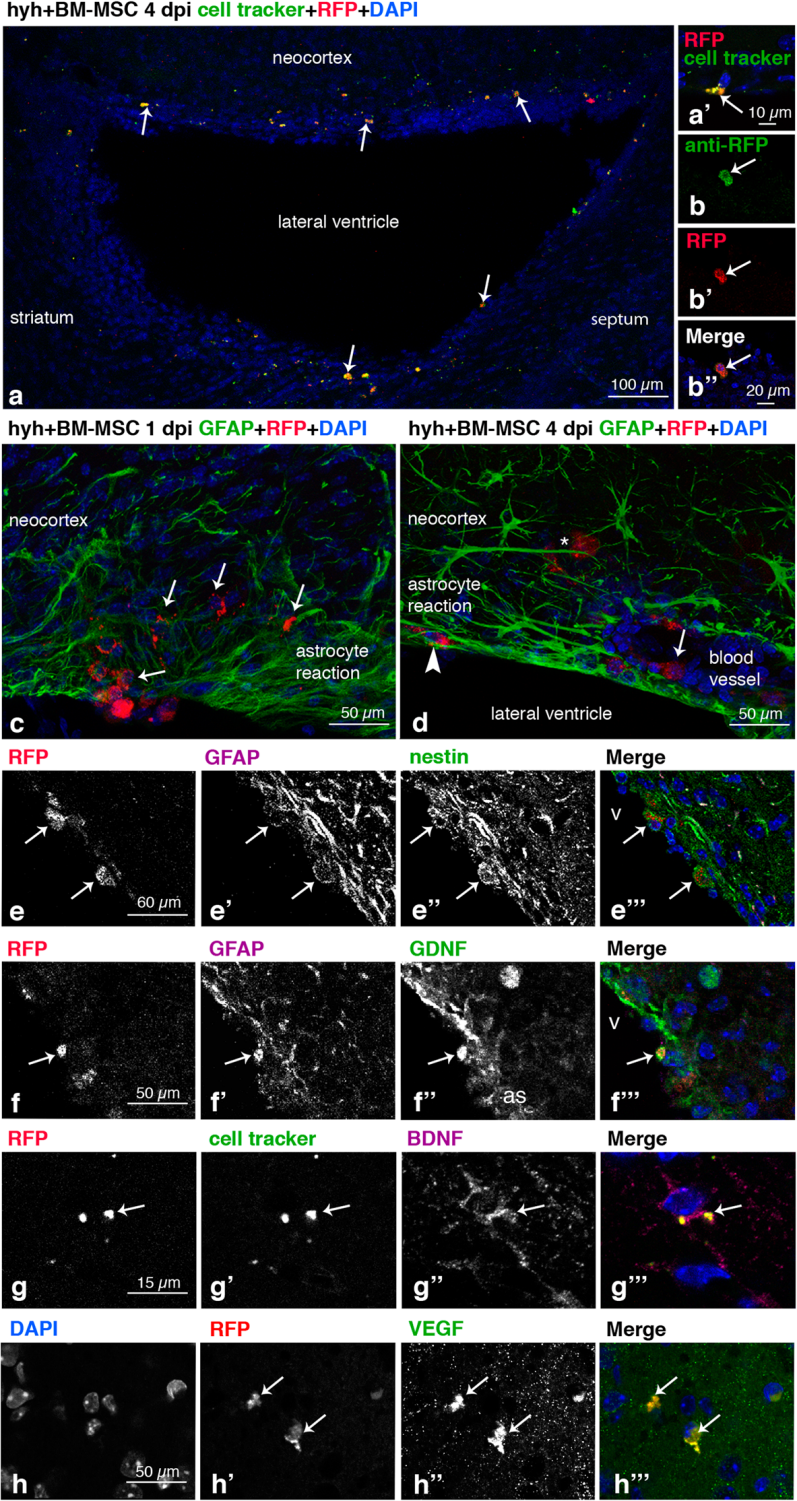
Location of BM-MSC in the hosting tissue and detection of neuroprotector factors expression. **a** Walls of the lateral ventricle of a hyh mouse administered at 20 days of age with BM-MSC expressing the mRFP1 (red) and labeled with a green cell tracker (white arrows), 4 days post-injection (dpi). **a’** Detail of a BM-MSC (RFP fluorescence, red) colabeled with the fluorescent green cell tracker (white arrow). **b**, **b”** Colabeling of the mRFP1 (red) with an antibody against RFP (green) in the administered BM-MSC, 4 dpi, in the neocortex of a hyh mouse injected at 20 days of age. **c** BM-MSC (red, white arrows) entering into the brain parenchyma of a hyh mouse 20 days of age, 1 dpi, through a ventricle surface presenting a loose periventricular layer of reactive astrocytes (GFAP immunolabeling, green) in the neocortex wall. **d** In the neocortex walls of hyh injected at P20, BM-MSC were found in three different locations at 4 dpi: between the dense layer of reactive astrocytes covering the ventricle surface (arrowhead, GFAP immunolabeling in green), around the blood vessels (arrow), and deep into the brain parenchyma (asterisk). **e**–**e”’** Coexpression in BM-MSC (mRFP1, red; arrows) at 4 dpi in the neocortex wall of GFAP (magenta) and nestin (green). **f**–**f”’** Coexpression at 4 dpi in BM-MSC (red mRFP1, arrow) of GFAP (magenta) and GDNF (green). **g**–**g”’** BM-MSC (mRFP1, red; arrow) colabeled with the green cell tracker and anti-BDNF (magenta) at 4 dpi. **h**–**h”’** Expression in BM-MSC (red, mRFP1; arrows) of VEGF (green) at 4 dpi

One day after injection, BM-MSC were confirmed to have entered the periventricular parenchyma at regions where astrocyte reaction was not compactly developed (Fig. 2c; for a description of these regions, see [40]). Excepting this confirmation, the whole results below are referred to mice analyzed 4 days after injection of the BM-MSC. Four days after intraventricular injection, BM-MSC were found integrated into the damaged areas of the periventricular parenchyma in both lateral ventricles in hyh mice (Fig. 2a). Because neocortical walls, and in particular the white matter, are the more affected areas in hydrocephalus of human and animal models [20, 21, 30, 41–43], the analysis of the results was focused into this region.

In fixed brain sections, BM-MSC were detected by red fluorescence from donor mRFP1 expression and green fluorescence from the cell tracker dye applied in vitro before injection (Fig. 2a), as well as immunolabeling with an antibody recognizing mRFP1 (Fig. 2b). Thus, 4 days after transplantation, BM-MSC were located in the neocortical walls within the layer of reactive astrocytes covering the denuded ependyma surface (for a description of these regions, see [20, 40, 44]), around the blood vessels, and intermixed with the diffuse reactive astrocytes in the white matter (Fig. 2d). Transplanted BM-MSC expressed the stem cell marker nestin (Fig. 2e) and also presented a weak expression of GFAP compared to neighboring reactive astrocytes (Fig. 2e). The BM-MSC were also found expressing the neuroprotector factors GDNF, BDNF, and VEGF in the same way as in vitro before injection (Fig. 2f–h).

### Effects on inflammatory conditions and glial reactions

**Fig. 3:**
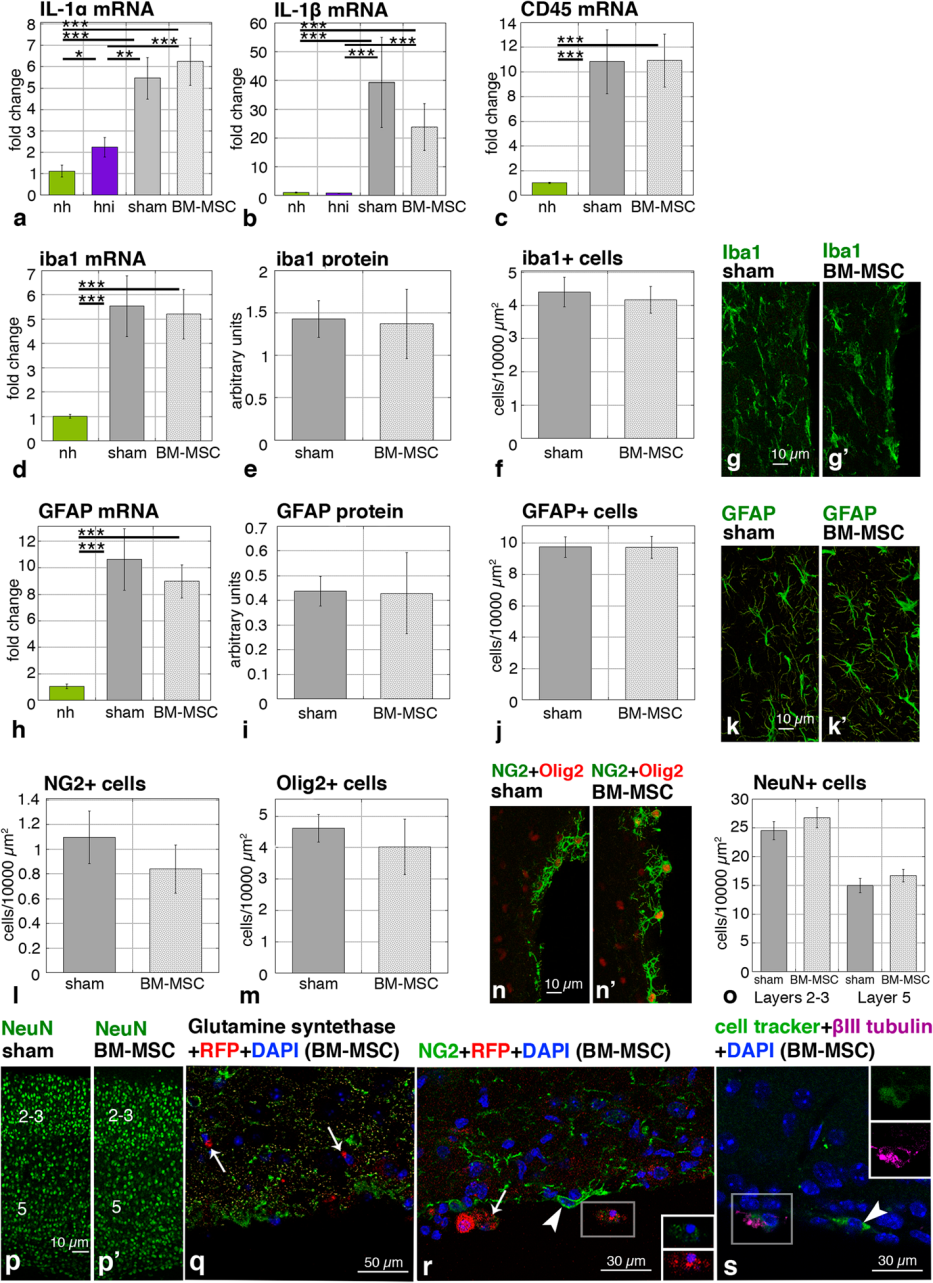
Levels of proinflammatory cytokines and densities of neural cells. **a**, **b** Levels of mRNA of interleukins IL-1α and IL-1β in the groups of mice: non-hydrocephalic (nh), hydrocephalic hyh non-injected (hni), hydrocephalic hyh sham-injected (sham), and hydrocephalic hyh BM-MSC-treated (BM-MSC). **c**–**f** Levels of mRNA, protein, and cell densities for CD45 and iba1 as microglia markers. **g**, **g’** Immunofluorescence for iba1 in the neocortex of a hydrocephalic hyh mouse treated with BM-MSC and in a hydrocephalic hyh sham-injected mouse. **h**–**j** Levels of mRNA, protein, and cell densities for the GFAP astrocyte marker. **k**, **k’** Immunofluorescence for GFAP in the neocortex of a hydrocephalic hyh mouse treated with BM-MSC and in a hydrocephalic hyh sham-injected mouse. **l**, **m** Densities of NG2+ and Olig2+ cells in tissue sections. **n**, **n’** Immunofluorescence for NG2 (green) and Olig2 (red). **o** Densities of NeuN+ cells in sections of the neocortical layers 2–3 and 5. **p**, **p’** Immunofluorescence for NeuN (green) in the neocortex. **q** Immunofluorescence for glutamine synthetase. The BM-MSC (mRFP1, red) present no reaction for glutamine synthetase (arrows). **r** Immunofluorescence for NG2. The BM-MSC present a weak immunoreaction (arrows) compared to NG2 cells (arrowheads). **s** Immunofluorescence for β-III tubulin (magenta) in BM-MSC (mRFP1, red) labeled with the green cell tracker in the neocortical wall. Arrowhead points to a β-III tubulin negative BM-MSC. Insets in **r** and **s** represent splitting of the channels showing immunolabeling for NG2 and cell tracker (green) and β-III tubulin (magenta) in the framed red fluorescent BM-MSC. *P < 0.05, ***P < 0.02, ***P < 0.01 Wilcoxon-Mann-Whitney test

In order to be a possible treatment, introduction of the stem cells should not induce extensive inflammatory responses in animals already compromised. Thus, to evaluate possible immunological rejection, the expression of inflammatory interleukins (IL-1α, IL-1β) was analyzed in four groups of mice: hydrocephalic hyh treated with BM-MSC, hydrocephalic hyh sham-injected, hydrocephalic hyh non-injected (hyh without any surgery), and non-hydrocephalic (without any surgery). Other cytokines such as the tumor necrosis factor alpha (TNFα) were not considered for analysis because a previous report in hyh mice indicated they would already be raised according to their etiopathology [43]. Levels of the interleukins were higher in BM-MSC-injected and sham-injected hydrocephalic hyh mice than in the non-hydrocephalic mice or non-injected hydrocephalic hyh mice (Fig. 3a, b). These results indicate that surgical procedure induces high IL levels. These results also suggest that non-injected animals are not an appropriated control to evaluate BM-MSC inflammatory response in the injected hydrocephalic hyh mice. However, we can efficiently compare hydrocephalic injected and non-injected hyh mice to determine inflammatory reaction in the presence of BM-MSC. Interleukin levels in hydrocephalic hyh mice with BM-MSC were not significantly different from hydrocephalic hyh sham-injected mice.

Levels of the anti-inflammatory interleukin IL-10 were negligible in all the groups of mice, including the BM-MSC treated one. Thus, in the present study and conditions, the presence of an anti-inflammatory effect cannot be concluded.

Microglia reaction was studied by expression of iba1 (mRNA and protein; microglia marker) and CD45 (mRNA; activated microglia marker). Hydrocephalic hyh mice (BM-MSC-treated and sham-injected) had upregulated CD45 and iba1 levels compared to non-hydrocephalic mice. In contrast, no differences in CD45 and iba1 levels were found between BM-MSC-injected and sham-injected mice (Fig. 3c–e). In the same way, the densities of iba1 immunoreactive cells in the neocortex were similar between hyh mice transplanted with BM-MSC and sham-injected (Fig. 3f, g). Activated microglia (CD45 labeling) or neutrophils (myeloperoxidase labeling) were not detected in the neighborhood of the BM-MSC. Analysis of astrocyte reactivity using the GFAP marker did not reveal any difference between the two groups of hydrocephalic hyh mice, BM-MSC-injected and sham-injected (Fig. 3h–k). The similar levels of Ki67 mRNA in the neocortex of hydrocephalic hyh mice treated with BM-MSC and sham-injected (Additional file 3) support the absence of glial reactions or uncontrolled proliferation of BM-MSC. Transplanted BM-MSC were not detected forming clusters but were found spared in the white matter and astroglial reactions.

### Effects of transplanted BM-MSC into the different neural cell populations

In order to confirm that BM-MSC do not transdifferentiate after transplantation, an immunohistochemical characterization of the integrated BM-MSC was carried out. In the neocortex, there was no increase in the densities of neural cell populations due to BM-MSC treatment, thus suggesting an absence of new neuronal cells or transdifferentiation of the BM-MSC. The densities of NG2+ (oligodendrocyte progenitors), Olig2+ (oligodendrocytes), and NeuN+ (mature neurons) cells were similar between hydrocephalic hyh mice treated with BM-MSC or sham injection (Fig. 3l–p). The density of mature neurons in the different neocortical layers was similar between both groups of hydrocephalic mice, treated with BM-MSC and sham-injected (Fig. 3o, p). Transplanted BM-MSC were immunonegative for the enzyme glutamine synthetase, which should only be present in astrocytes (Fig. 3q). BM-MSC were weakly labeled with anti-NG2, unlike oligodendrocyte progenitors that exhibited strong immunolabeling (Fig. 3r). A few BM-MSC were weakly stained with β-III tubulin (Fig. 3s), in contrast to the strongly labeled neuroblasts. These patterns of immunostaining of the BM-MSC integrated into the brain parenchyma resembled the state of these cells in vitro before injection (Fig. 1m).

### Reduction of apoptosis

**Fig. 4:**
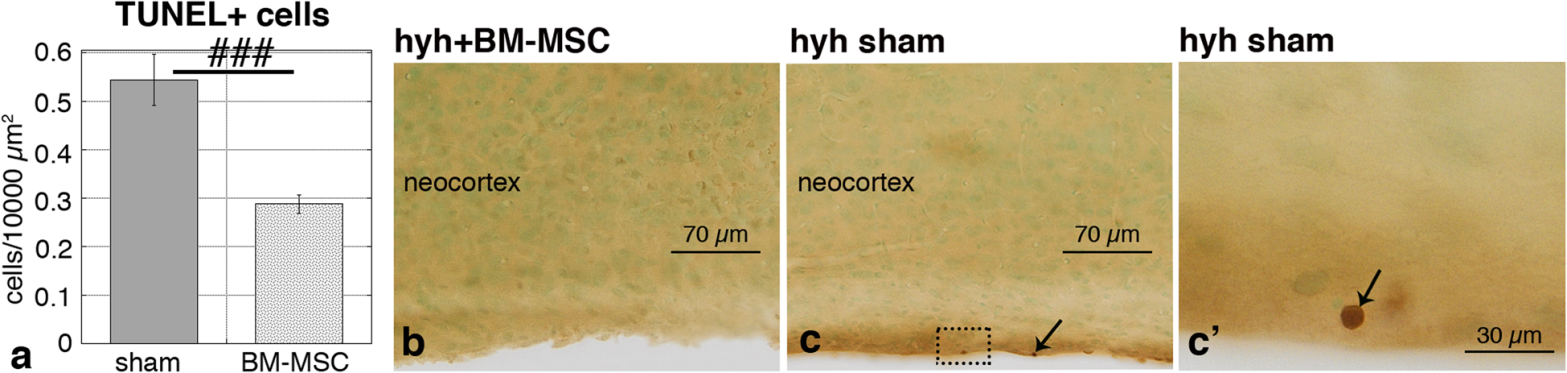
Densities of apoptotic cells in the neocortex. **a** TUNEL+ cells in hydrocephalic hyh mice treated with BM-MSC and hydrocephalic hyh sham-injected mice, 4 dpi. ^###^*P* < 0.01 Student’s *t* test. **b**, **c** Periventricular apoptotic cells (*arrows*) in a hyh mouse administered with BM-MSC cells and in a hydrocephalic hyh sham-injected mouse. **c’** Detail of an apoptotic cell shown in the area framed in **c**

Because BM-MSC have been associated with neuroprotective roles [13, 45], the density of apoptotic cells in host tissues was analyzed by TUNEL labeling. In hydrocephalic hyh mice treated with BM-MSC, the number of apoptotic cells was reduced by 50% compared to sham-injected hyh mice (Fig. 4a). In both cases, the location of apoptotic cells as seen by TUNEL+ chromogenic labeling was mainly observed within the damaged periventricular surfaces (Fig. 4b, c).

### Reduction in the levels of osmolytes

**Fig. 5:**
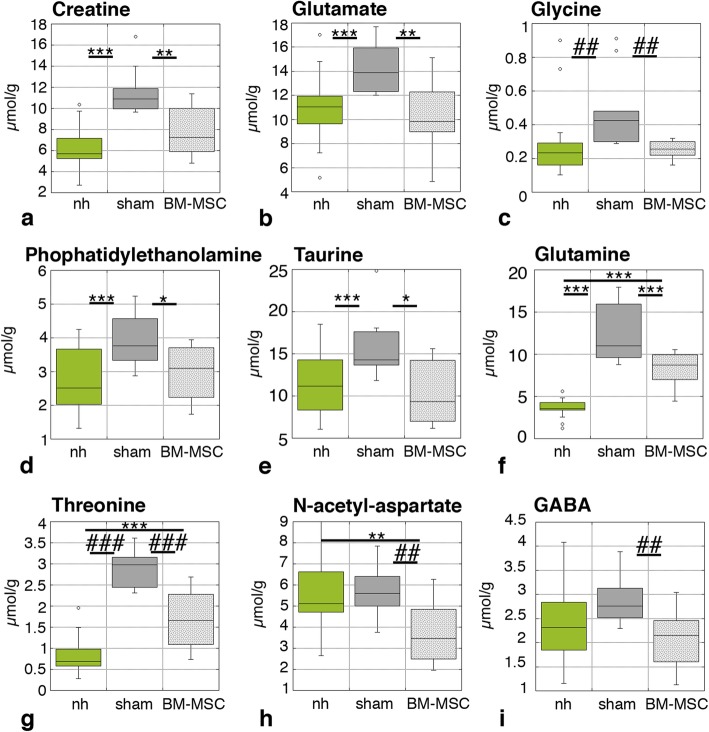
Levels of metabolites in the neocortex. Analysis was carried out in non-hydrocephalic mice, hydrocephalic hyh mice transplanted with BM-MSC, and hydrocephalic hyh sham-injected mice, 4 dpi. *P < 0.05, **P < 0.02, ***P < 0.01 Wilcoxon-Mann-Whitney test; ^##^*P* < 0.02, ^###^*P* < 0.01 Student’s *t* test

Possible recovery of the neocortical tissue was investigated by analyzing the metabolic state of the neocortex by ^1^H HR-MAS NMR spectroscopy in ex vivo samples. Many metabolites are significantly increased under conditions of congenital hydrocephalus in hyh mice and indicate pathological severity [30]. Metabolites/osmolytes to be studied were selected based on standard human clinic NMR practices and according to a previous study in the hyh mouse [30]. Four days after BM-MSC implantation, creatine, glutamate, glycine, phosphatidylethanolamine, and taurine were found at lower concentrations in the hydrocephalic hyh transplanted with BM-MSC compared to hydrocephalic hyh mice sham-injected mice (Fig. 5a–e). Interestingly, BM-MSC treated mice exhibited recovered metabolite levels similar to non-hydrocephalic mice (Fig. 5a–e). While levels of glutamine and threonine were also reduced in hydrocephalic hyh mice treated with BM-MSC compared to hydrocephalic hyh sham-injected mice (Fig. 5f, g), they did not recover entirely back to non-hydrocephalic levels. In the case of *N*-acetyl-aspartate and GABA, while hydrocephalic hyh mice exhibited significantly increased levels, the BM-MSC treatment still reduced the levels of these metabolites (Fig. 5h, i). Other metabolites did not present significant differences in their levels according to the trends described above (Additional file 4). Logistic regression analysis for the discrimination of both groups of hydrocephalic hyh mice (BM-MSC-injected and sham-injected) showed that the combination of creatine, glutamate, *N*-acetyl-aspartate, taurine, and threonine had the highest predictive score that allows correct discrimination between the two groups of hydrocephalic hyh mice, treated with BM-MSC and sham-injected (Table 2).

**Table 2:** Logistic regression analysis for the discrimination between hyh mice treated with BM-MSC and hydrocephalic sham-injected mice

Metabolite	*B*	Standard error	Wald	df	*P* value*	*eB*	Lower	Upper
Creatine	0.972	0.489	3.961	1	0.047*	2.644	1.015	6.891
Glutamate	0.657	0.300	4.803	1	0.028*	1.929	1.072	3.471
*N*-acetyl-aspartate	1.086	0.496	4.781	1	0.029*	2.961	1.119	7.835
Threonine	3.989	1.965	4.121	1	0.042*	54.007	1.147	2542.003
Taurine	0.467	0.226	4.249	1	0.039*	1.595	1.023	2.485

For each metabolite, the following are shown: *B*, equation coefficient; standard error; Wald-test statistics; df, degrees of freedom; *P* value; exponential of *B*; and the lower and upper confident intervals

*P value significant

### In vitro cytokine secretion from BM-MSC stimulated with TNFα

**Fig. 6:**
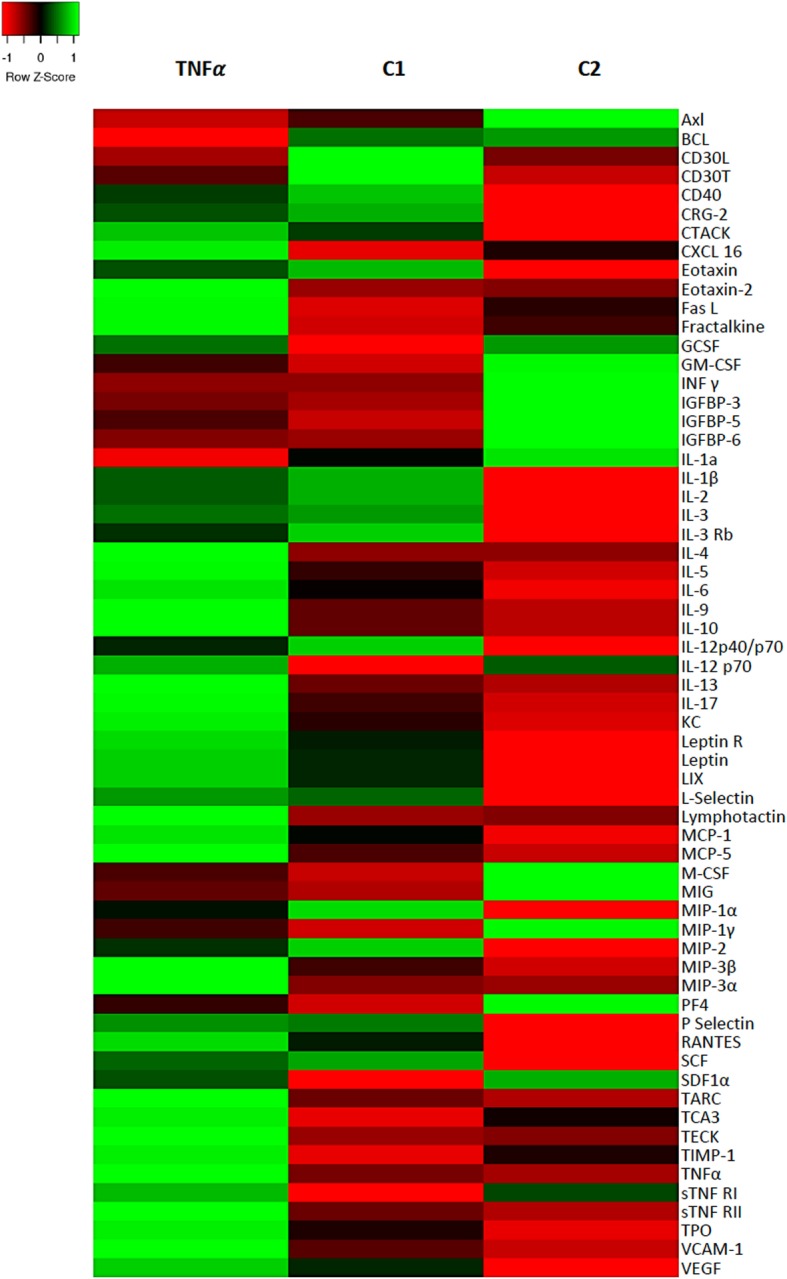
Heat map showing the relative concentrations of cytokines secreted by BM-MSC stimulated with TNFα. C1, control without TNFα. C2, equal to C1 with the addition of 10% FBS. Cytokines were detected using an array. Colors were assigned according to the relative scale of expression. *Z* = 0 represents the mean value

To elucidate the mechanisms that could be associated with BM-MSC integration into the astrocyte reaction and tissue recovering, an in vitro analysis was performed after incubation of the BM-MSC with TNFα. Our previous studies have proved that there are high levels of TNFα in the periventricular astrocyte reaction in hyh mice [43]. Thus, the secretome of BM-MSC under the presence of TNFα was investigated. VEGF and several cytokines were found upregulated under TNFα incubation (Fig. 6, Tables 3 and 4, Additional file 5). According to the database, such upregulation can be implicated in angiogenesis, endothelial permeability, locomotion, cell movement, and the orchestration of inflammatory and immune responses.

**Table 3:** Over-represented biological processes of the BM-MSC secretome under TNFα compared with control C1

Biological process	TNFα (*P* value)	Control C1 (*P* value)	ID (term GO)
Positive regulation of GTPase activity	1.76 × 10^−20^	2.65 × 10^−01^	GO:0043547
Cellular response to tumor necrosis factor	2.75 × 10^−22^	3.52 × 10^−01^	GO:0071356
Granulocyte chemotaxis	1.49 × 10^−27^	1.04 × 10^−09^	GO:0071621
Positive regulation of ERK1 and ERK2 cascade	3.34 × 10^−19^	4.51 × 10^− 01^	GO:0070374
Cytokine-mediated signaling pathway	6.74 × 10^−29^	1.32 × 10^−07^	GO:0019221
Inflammatory response	1.88 × 10^−24^	1.28 × 10^−03^	GO:0006954
Antimicrobial humoral immune response mediated by antimicrobial peptide	6.43 × 10^−03^	–	GO:0061844
Cellular response to lipopolysaccharide	1.18 × 10^−02^	3.24 × 10^−04^	GO:0071222
MAPK cascade	3.42 × 10^−10^	3.45 × 10^−02^	GO:0000165
Innate immune response	7.55 × 10^−09^	–	GO:0045087

Analysis of data using the PANTHER classification system obtained with a cytokine array in the culture media. The corresponding *P* value is shown according to the binomial test. The identity (ID) of the gene products (GO terms from PANTHER GO slim) describing the function is indicated

**Table 4:** Upregulated proteins secreted by BM-MSC under TNFα stimulation compared with control C1

Protein	Biological processes	Abundance ratio (sample/Control C1)	*P* value
C-X-C motif chemokine 3	1, 2, 3, 4, 5	11.38	1 × 10^−1^
C-type lectin domain family 4 member E	3,4, 5, 6	8.464	1 × 10^−17^
Platelet factor 4	1, 3, 4, 7	5.818	5.54 × 10^−10^
C-X-C motif chemokine 10	1, 3, 4, 5	5.198	2.44 × 10^−12^
Pentraxin-related protein PTX3	3, 4, 5	4.613	5.66 × 10^−11^
Stromelysin-1	4, 5, 8	3.936	4.98 × 10^−09^
Isoform 2 of sequestosome-1	4, 5, 6, 7, 8, 9, 10	3.842	9.45 × 10^−09^
Neutrophil gelatinase-associated lipocalin	3, 4, 5, 7, 9, 10, 11	3.267	0.0002
CD82 antigen	4, 5	2.869	3.98 × 10^−05^
Tumor necrosis factor receptor superfamily member 1B	3, 4, 5, 12, 13	2.762	0.0006
Copper transport protein ATOX1	2, 5, 11	2.572	7.45 × 10^−05^
Prostaglandin G/H synthase 2	3, 4, 5, 6, 8	2.421	0.005
Intercellular adhesion molecule 1	1, 2, 3, 4, 5, 7, 9	2.107	0.002
GRIP1-associated protein 1	4, 8, 11	2.023	0.02
MAGUK p55 subfamily member 6	8	2.013	0.03

Mass spectrometry analysis of secretome. The *P* value is calculated in a background-based ANOVA. This method uses the background population of ratios for all peptides and proteins to determine whether any given single peptide or protein is significantly changing relative to that background. Biological processes: 1, cellular component movement; 2, cellular homeostasis; 3, defense response; 4, regulation of biological process; 5, response to stimulus; 6, cell differentiation; 7, cell organization and biogenesis; 8, metabolic process; 9, cell communication; 10, cell death; 11, transport; 12, cell proliferation; 13, development. More data of these proteins is shown in Additional file 5

## Discussion

### Integration of BM-MSC into the damaged periventricular walls without rejection

BM-MSC transplanted into the ventricles of hydrocephalic hyh mice exhibited successful migration toward sites of damaged brain tissue and dispersed into the neocortex periventricular zones that were presenting ischemia, interstitial edema, axonal damage, and astrocyte reactivity [4, 20, 41, 46, 47]. The BM-MSC presumably were attracted by chemokines [13, 48] and TNFα [49], the latter of which has been proposed to be upregulated and released by the periventricular reactive astrocytes in the hyh mouse [43].

In young, healthy mice, it has been reported that MSC administered intracerebroventricularly form cell clusters attached to the ventricle walls [50]. However, in the present study, BM-MSC never formed clusters. In contrast, they were found spread throughout the cerebral ventricle walls. Two explanations can be given for such a difference: first, the five times smaller number of MSC (200,000/animal) applied in the present investigation compared with Jungwirth’s study (1,000,000/animal); secondly, the pathological environment present in the brain of the hydrocephalic hyh mouse. In any case, the number of cells applied in the present study is sufficient to produce favorable effects in severe hydrocephalus, without forming such clusters or adverse effects. Nevertheless, the number of cells to transplant should be taken into account for the clinical application of MSC.

MSC produce factors that affect the maturation and function of immune cells, suppressing innate and adaptive immunity [49]. However, in a study with BM-MSC injected into the parenchymal tissue of mature, healthy rats, stem cells have been found missed after 14 days, which was interpreted as a rejection [51]. It must be considered that MSC would either suppress or promote inflammation according to the milieu where they are applied [49]. Thus, anti-inflammatory effects would take place during the late phase of inflammation [49]. In the present study, the presence of inflammatory conditions in hyh mice before transplantation with BM-MSC (present results, see in [43]) would support the absence of rejection. The absence of rejection in treated hyh mice is evidenced by the lack of recruited neutrophils, macrophages, or activated microglia in the areas where BM-MSC are integrated 4 days after injection. Besides, there was no increase in the astrocyte reaction at this time.

### Absence of transdifferentiation of the transplanted BM-MSC

There is controversy regarding the possibility of the transdifferentiation of MSC into neural cells [52]. It has been suggested that the host environment of the developing brain allows the transdifferentiation of MSC [52, 53]. Also, MSC have been suggested capable of stimulating neurogenesis and formation of new astrocytes or oligodendrocytes [54, 55]. In the present study, BM-MSC were integrated into the periventricular walls of hyh mice keeping the expression pattern for nestin, GFAP, NG2, β-III tubulin, Olig2, and NeuN. Furthermore, the molecular and cellular analysis in hydrocephalic hyh mice treated with BM-MSC did not reveal any increase of neural cells. Therefore, the present results have shown that injected BM-MSC remained as stem cells without undergoing into transdifferentiation.

### Neuroprotective effect of BM-MSC in hydrocephalic mice

In hydrocephalic hyh mice treated with BM-MSC, the reduction in the number of apoptotic cells, and the decreased levels of neurocytotoxic metabolites and osmolytes suggest the induction of a neuroprotective environment. As discussed above, the protection would exclude cell replacement. Therefore, the effect could be through the production of growth factors or cytokines, vascular effects, reduction of oxidative stress, and modulation of the inflammatory response, as it has been described in other experimental treatments [11, 15, 50].

Because a heterogeneous population has been transplanted to hydrocephalic hyh mice, more than one effect is expected. In this way, 10% of MSC were positive for CD11b, a molecule that inhibits TLR-induced inflammatory responses by the inhibition of inflammatory cytokines (IL-6 and TNFα) and increasing anti-inflammatory cytokine (IL-10 and TGF-β) production in microglia [56]. This suggests that part of the transplanted cells could have an anti-inflammatory role.

In addition, in the present investigation, it has been found that transplanted BM-MSC express the neurotrophic factors GDNF, BDNF, NGF, and VEGF, which have been implicated in increasing neuronal survival and in reducing apoptosis [13, 15, 48, 57–61]. The production of these factors could also be increased in hypoxic conditions [62], which could be present in the hydrocephalic hyh mice with high intracranial pressure [30]. BDNF secreted by MSC could also protect neurons from glutamate excitotoxicity by reducing neuronal sensitivity to glutamate [63]. However, it is unlikely that bioactive factors released by local BM-MSC per se were the only and direct contributors to the improvement of neurological deficits [48]. Interestingly, BM-MSC could activate astrocytes to release neurotrophic factors, such as GDNF or TNFα, which would promote and facilitate tissue recovery [48, 60]. TNFα could also stimulate the production of paracrine factors such as VEGF [62]. TNFα has been found produced by periventricular reactive astrocytes in the hyh mouse and human fetuses with hydrocephalus [43]. VEGF and GDNF expressions have been also found in these reactive astrocytes (unpublished observations, and see GDNF immunostaining in Fig. 2f). VEGF is one of the most effective trophic factors inducing angiogenesis, thus contributing to the recovery of ischemic conditions [64–66]. The secretion of these neurotrophic factors by BM-MSC and the neighbor reactive astrocytes could create an optimal environment. Further studies are needed to know the mechanisms behind the action of the neurotrophic factors.

In the present study, the secretome analysis of BM-MSC licensed with TNFα points to some mechanisms that can be implicated in the brain parenchyma recovering. In this way, lipocanin-2 has been described stimulated by TNFα and involved in anti-inflammatory responses [67]. ATOX1 is anti-oxidant and anti-inflammatory [68]. Pentraxin-3 has also been found upregulated in our experiments. Previous studies described Pentraxin-3 as a factor that promotes recovery in neural tissue after ischemia [69]. The secretion of the TIM-1 and CXCL16 has been found implicated in tissue recovery mediated by endothelial cells [70]. VEGF has also been proved implicated in tissue recovery mediated by increased permeability of the blood-brain barrier [71]. Therefore, the secretion of several proteins was upregulated after TNFα stimulation. These proteins could cause neuroprotection and explain the positive effects on the metabolite levels found in the present investigation discussed below.

### Recovering metabolite concentrations in the neocortex of hydrocephalic hyh mice treated with BM-MSC

Several metabolites are present in high concentrations in the neocortex of hydrocephalic hyh sham-injected mice, probably as a consequence of periventricular edema, ischemia, and degenerative conditions [30]. These levels reported here are similar to those previously described in hydrocephalic hyh mice with severe hydrocephalus in the absence of surgery or treatment [30]. Comparatively, the levels of these metabolites in hydrocephalic hyh mice treated with BM-MSC for 4 days are reduced enough to resemble those of non-hydrocephalic mice.

Metabolite/osmolyte reduction can represent a recovering in the environment of the damaged neocortex. One of the significant metabolites is creatine, which has been related to neuroprotective effect against ischemic damage as phosphocreatine allows ATP synthesis in the absence of glucose or oxygen [72]. Another key metabolite is glutamate. In astrocytes, through glutamate dehydrogenase, glutamate contributes to the tricarboxylic cycle intermediate α-ketoglutarate and ATP production after brain ischemia [73]. Extracellular presence of glutamate is excitotoxic since it provokes the activation of glutamate receptors in the post-synaptic membrane, leading to the destruction of the calcium buffer system, mitochondria damage, and inhibition of phosphatidylcholine-specific phospholipase C [74]. High levels of glutamate in the interstitial fluid can be a consequence of neural or glial destruction [74]. In hydrocephalic hyh mice treated with BM-MSC, glycine, phosphatidylethanolamine, taurine, and threonine, which are considered osmoregulatory metabolites [75, 76], were found following the same trends as glutamine and glutamate. Phosphatidylethanolamine and taurine are regulatory osmolytes that have been related to brain injury [77]. *N*-acetyl-aspartate is a marker of neuronal activity that is usually used to determine neuronal density in NMR spectroscopy. In hydrocephalic hyh mice treated with BM-MSC, the levels of *N*-acetyl-aspartate are lower than in hydrocephalic hyh sham-injected mice. However, the analysis of neuronal density in the neocortex of both groups of hydrocephalic hyh mice has shown no difference. *N*-acetyl-aspartate is also considered an osmolyte required for myelination [78], and their lower levels in hydrocephalic hyh mice treated with BM-MSC could be a consequence of clearance in the periventricular edema.

## Conclusions

BM-MSC can integrate into the neocortical walls of the lateral ventricles of hydrocephalic hyh mice with severe hydrocephalus without rejection, remaining undifferentiated and integrated among reactive astrocytes in the periventricular regions. The neocortical tissue, after the BM-MSC therapy, showed signs of recovery, detected as a reduction of apoptosis and in reduced levels of hydrocephalic-associated metabolites/osmolytes. The experimental approach, including analysis of metabolite spectra with NMR, used in this work makes these findings transferable to the human clinic. Therefore, administration of BM-MSC seems to be a promising stem cell therapy for the treatment of neurodegenerative conditions present in severe obstructive hydrocephalus.

## Supplementary information

**Additional file 1.** BM-MSC immunophenotype. Immunophenotype profiles of unfixed BM-MSC for CD75, CD90, and CD45 markers by flow cytometry.

**Additional file 2.** BM-MSC immunophenotype. Immunophenotype profiles of unfixed BM-MSC for CD11b and F4/80 by flow cytometry.

**Additional file 3.** Ki67 levels. a. Levels of Ki67 mRNA in the neocortex of non-hydrocephalic mice (nh), hydrocephalic hyh mice transplanted with BM-MSC, and hydrocephalic hyh sham-injected mice, 4 days post-injection (dpi). b, c. Immunofluorescence for Ki67 (green) and GFAP (red, astrocyte labeling) in the neocortex of a hydrocephalic hyh mouse treated with BM-MSC and of a hydrocephalic hyh sham-injected mouse. Nuclear staining with DAPI (blue).

**Additional file 4 **: Metabolites in the neocortical tissue. Levels of metabolites recorded by HR-MAS in the neocortex of non-hydrocephalic mice (nh), hydrocephalic hyh mice transplanted with BM-MSC, and hydrocephalic hyh sham-injected mice. ***P < 0.01 Wilcoxon-Mann-Whitney test; ###*P* < 0.05, ##*P* < 0.02, ###P < 0.01 Student’s t-test.

**Additional file 5.** : Mass spectrometry analysis of the BM-MSC secretome under TNFα stimulation. Sum PEP Score corresponds to the score calculated on the basis of the posterior error probability (PEP) values of the peptide spectrum matches (PSM). Sum PEP Score indicates the probability that an observed PSM is incorrect. Molecular weights (MW) of the proteins are shown. Number of peptides and percentage of the identified proteins are indicated.

## Data Availability

The datasets used and/or analyzed during the current study are available from the corresponding author on reasonable request.

## Abbreviations

Axl: Tyrosine-protein kinase receptor UFO
BCL: Apoptosis regulator Bcl-2
BDNF: Brain-derived neurotrophic factor
BM-MSC: Bone marrow-derived mesenchymal stem cells
CD30L: Tumor necrosis factor ligand superfamily member 8
CD30T: Tumor necrosis factor receptor superfamily member 8
CD40: Tumor necrosis factor receptor superfamily member 5
CRG-2: C-X-C motif chemokine 10
CTACK: C-C motif chemokine 27
CXCL 16: C-X-C motif chemokine 16
DAPI: 4′,6-Diamidino-2-phenylindole
DMEM: Dulbecco’s modified Eagle’s medium
dpi: Days post-injection
EDTA: Ethylenediaminetetraacetic acid
Eotaxin-2: C-C motif chemokine 24
Fas L: Tumor necrosis factor ligand superfamily member 6
FBS: Fetal bovine serum
FGF: Fibroblast growth factor
GAPDH: Glyceraldehyde-3-phosphate dehydrogenase
GCSF: Granulocyte colony-stimulating factor
GDNF: Glial-derived neurotrophic factor
GFAP: Glial fibrillary acidic protein
GM-CSF: Granulocyte-macrophage colony-stimulating factor
HIF-1α: Hypoxia-inducible factor 1-alpha
^1^H HR-MAS NMR: ^1^H High-Resolution Magic Angle Spinning Nuclear Magnetic Resonance
hyh: Hydrocephalus with hop gait
Iba1: Ionized calcium binding adapter molecule 1
IGFBP-3: Insulin-like growth factor-binding protein 3
IGFBP-5: Insulin-like growth factor-binding protein 5
IGFBP-6: Insulin-like growth factor-binding protein 6
IL-1α: Interleukin-1 alpha
IL-1β: Interleukin-1 beta
IL-2: Interleukin-2
IL-3: Interleukin-3
IL-3 Rb: Interleukin-3 receptor class 2 subunit beta
IL-4: Interleukin-4
IL-5: Interleukin-5
IL-6: Interleukin-6
IL-9: Interleukin-9
IL-10: Interleukin-10
IL-12 p40/p70: Interleukin-12 subunit beta
IL-12 p70: Interleukin-12 subunit alpha
IL-13: Interleukin-13
IL-17: Interleukin-17A
INF γ: Interferon gamma
KC: Growth-regulated alpha protein
Leptin R: Leptin receptor
LIX: C-X-C motif chemokine 5
MCP-1: C-C motif chemokine 2
MCP-5: C-C motif chemokine 12
M-CSF: Macrophage colony-stimulating factor 1
MIG: C-X-C motif chemokine 9
MIP-1α: C-C motif chemokine 3
MIP-1γ: C-C motif chemokine 9
MIP-2: C-X-C motif chemokine 2
MIP-3α: C-C motif chemokine 20
MIP-3β: C-C motif chemokine 19
mRFP1: Monomeric red fluorescent protein 1
MSC: Mesenchymal stem cells
nh: Non-hydrocephalic mouse
nih: Non-injected hydrocephalic mouse
NG2: Neuronal/glial antigen 2
NGF: Nerve growth factor
PB: Phosphate buffer
PBS: Phosphate-buffered saline
PF-4: Platelet factor 4
SCF: Kit ligand
SDF-1α: Stromal cell-derived factor 1
αSNAP: *N*-ethylmaleimide-sensitive factor attachment protein alpha
sTNF RI: Tumor necrosis factor receptor superfamily member 1A
sTNF RII: Tumor necrosis factor receptor superfamily member 1B
TARC: C-C motif chemokine
TCA-3: C-C motif chemokine 1
TECK: C-C motif chemokine 25
TGF-β: Transforming growth factor beta
TIMP-1: Metalloproteinase inhibitor 1
TNFα: Tumor necrosis factor alpha
TPO: Thyroid peroxidase
VCAM-1: Vascular cell adhesion protein 1
VEGF: Vascular endothelial growth factor
