# Supplementary figures and images for: Neocortical tissue recovery in severe congenital obstructive hydrocephalus after intraventricular administration of bone marrow-derived mesenchymal stem cells
Source: Stem Cell Res Ther. 2020 Mar 17;11:121. doi: 10.1186/s13287-020-01626-6 (PMC7079418; doi:10.1186/s13287-020-01626-6)

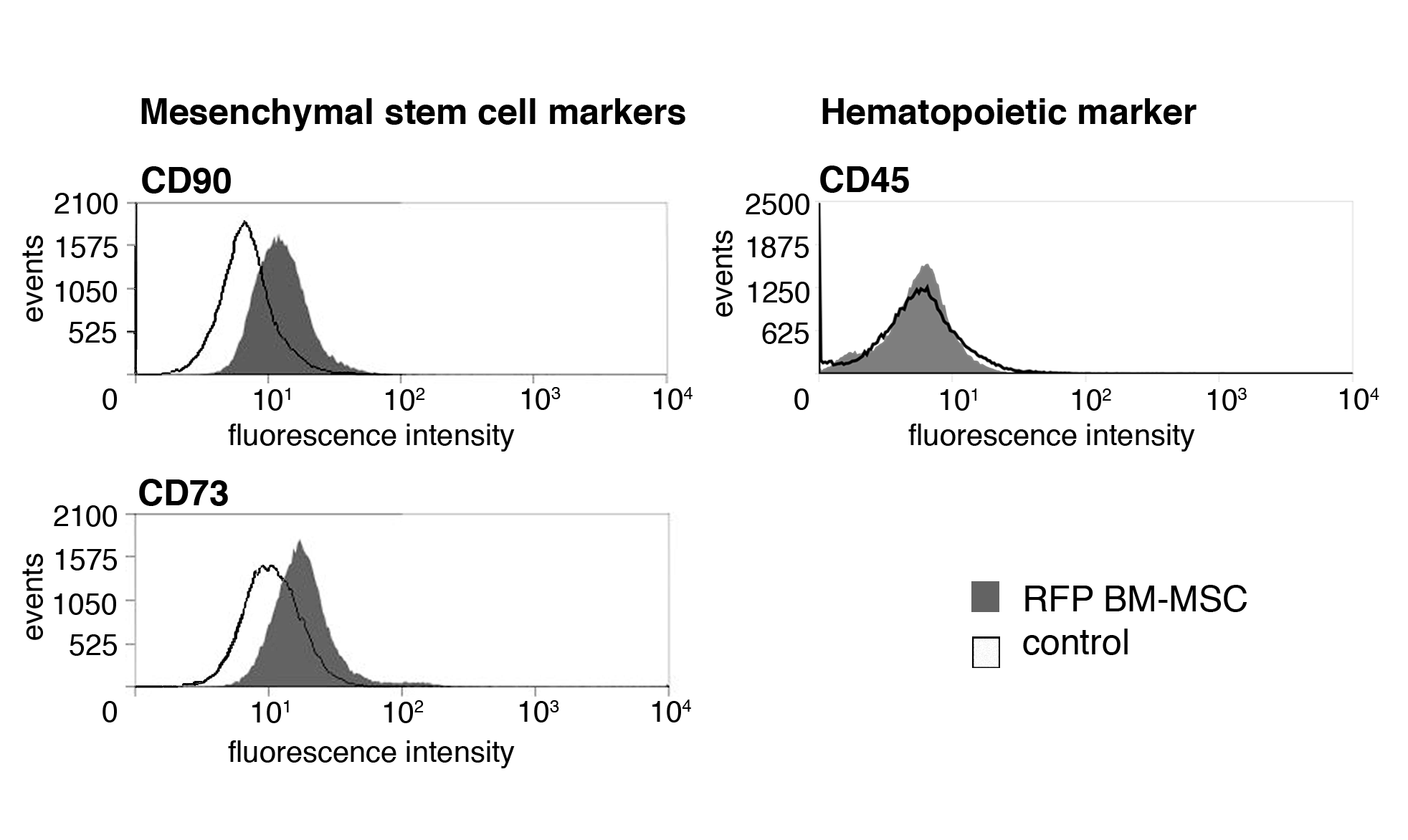

Supplement: Supplementary file 1 — Additional file 1. BM-MSC immunophenotype. Immunophenotype profiles of unfixed BM-MSC for CD75, CD90, and CD45 markers by flow cytometry. [file 13287_2020_1626_MOESM1_ESM.tif]

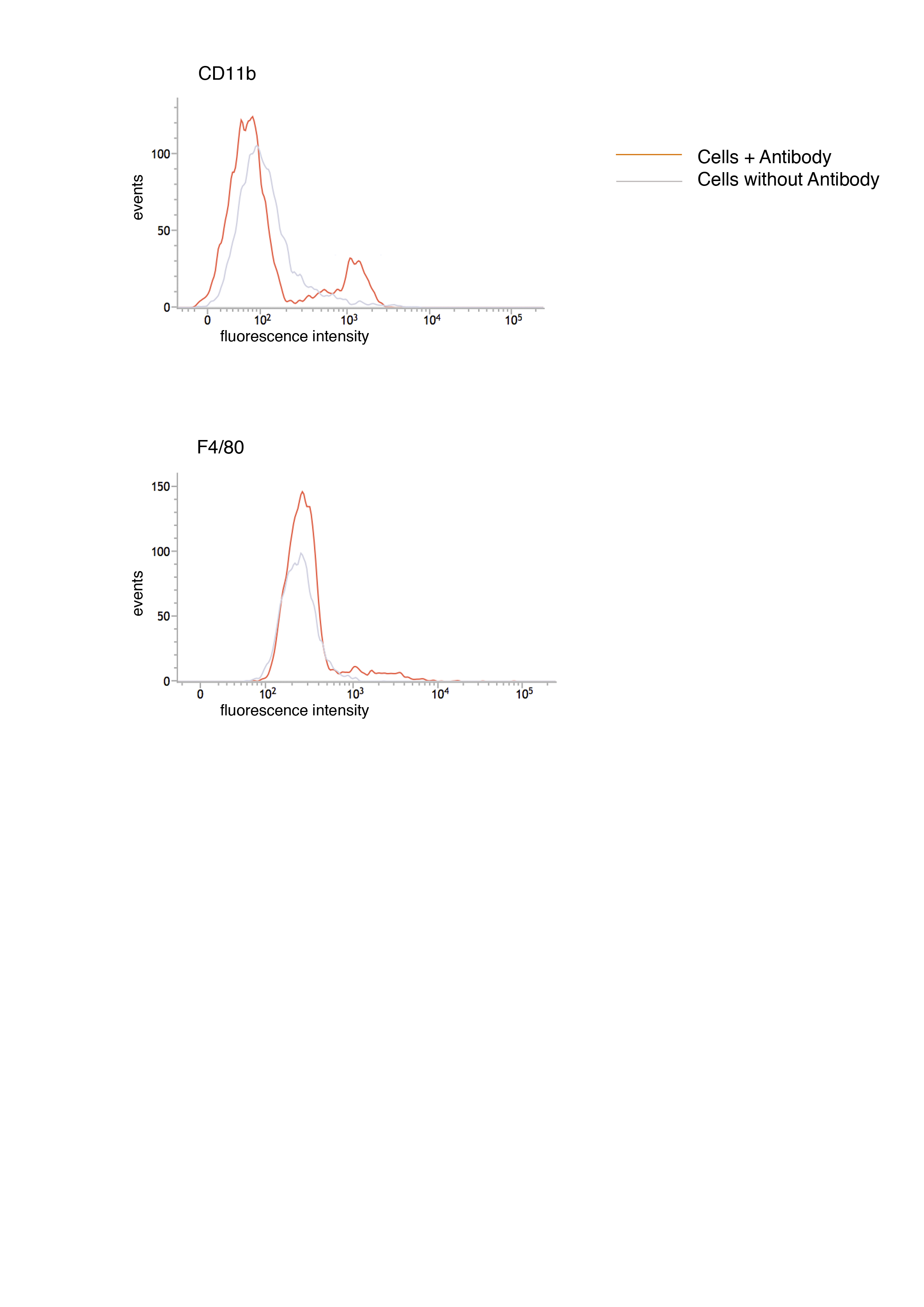

Supplement: Supplementary file 2 — Additional file 2. BM-MSC immunophenotype. Immunophenotype profiles of unfixed BM-MSC for CD11b and F4/80 by flow cytometry. [file 13287_2020_1626_MOESM2_ESM.tif]

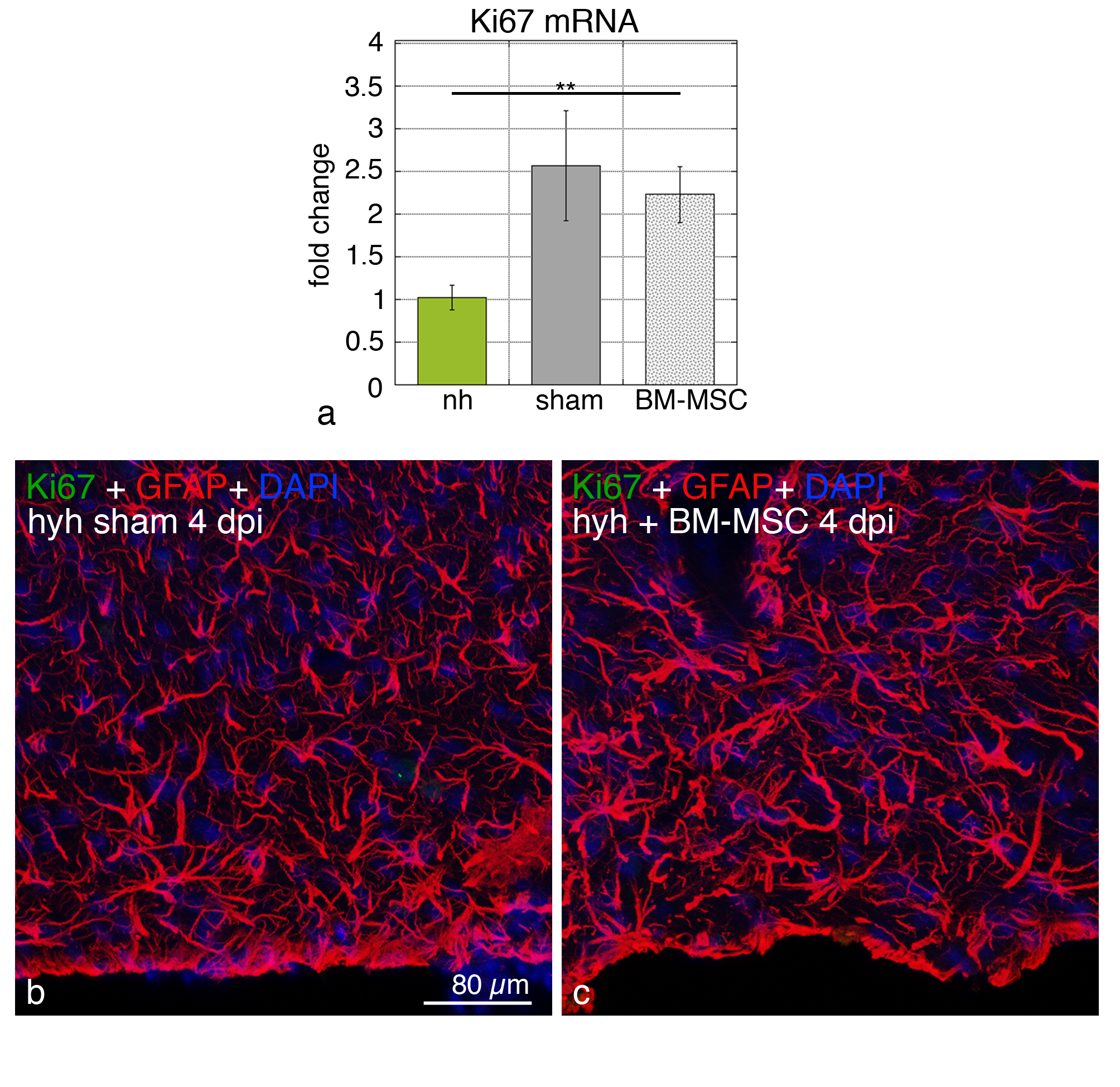

Supplement: Supplementary file 3 — Additional file 3. Ki67 levels. a. Levels of Ki67 mRNA in the neocortex of non-hydrocephalic mice (nh), hydrocephalic hyh mice transplanted with BM-MSC, and hydrocephalic hyh sham-injected mice, 4 days post-injection (dpi). b, c. Immunofluorescence for Ki67 (green) and GFAP (red, astrocyte labeling) in the neocortex of a hydrocephalic hyh mouse treated with BM-MSC and of a hydrocephalic hyh sham-injected mouse. Nuclear staining with DAPI (blue). [file 13287_2020_1626_MOESM3_ESM.tif]

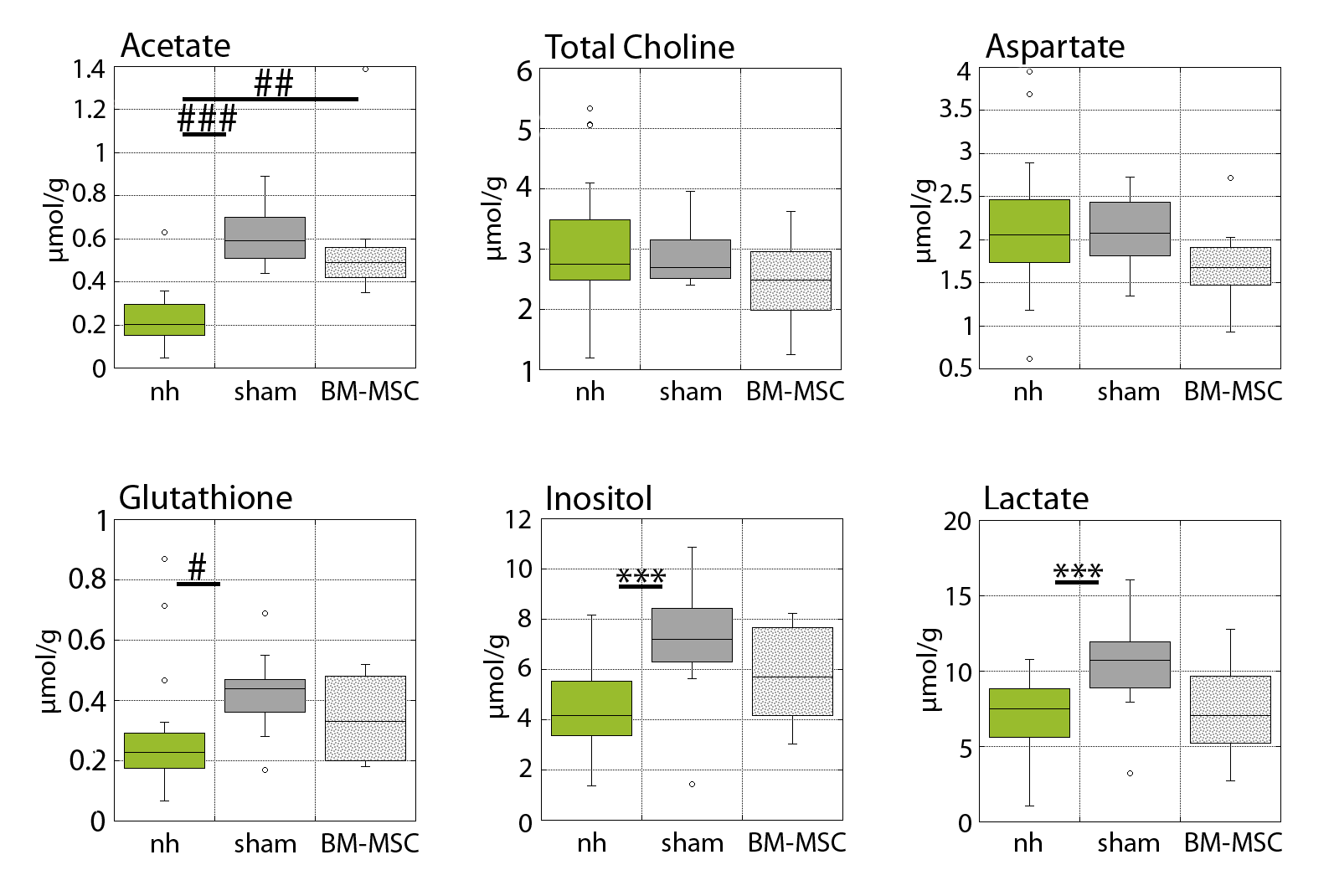

Supplement: Supplementary file 4 — Additional file 4 : Metabolites in the neocortical tissue. Levels of metabolites recorded by HR-MAS in the neocortex of non-hydrocephalic mice (nh), hydrocephalic hyh mice transplanted with BM-MSC, and hydrocephalic hyh sham-injected mice. ***P < 0.01 Wilcoxon-Mann-Whitney test; ###P < 0.05, ##P < 0.02, ###P < 0.01 Student’s t-test. [file 13287_2020_1626_MOESM4_ESM.tif]
