# Supplementary material for: Neocortical tissue recovery in severe congenital obstructive hydrocephalus after intraventricular administration of bone marrow-derived mesenchymal stem cells
Source: Stem Cell Res Ther. 2020 Mar 17;11:121. doi: 10.1186/s13287-020-01626-6 (PMC7079418; doi:10.1186/s13287-020-01626-6)
Supplement: Supplementary file 5 — Additional file 5. : Mass spectrometry analysis of the BM-MSC secretome under TNFα stimulation. Sum PEP Score corresponds to the score calculated on the basis of the posterior error probability (PEP) values of the peptide spectrum matches (PSM). Sum PEP Score indicates the probability that an observed PSM is incorrect. Molecular weights (MW) of the proteins are shown. Number of peptides and percentage of the identified proteins are indicated. [file 13287_2020_1626_MOESM5_ESM.docx]

| Protein description | Gene name | Sum PEP Score | Protein coverage (%) | Number of peptides | MW (kDa) |
| --- | --- | --- | --- | --- | --- |
| C-X-C motif chemokine 3 | Cxcl3 | 11.055 | 22 | 2 | 10.7 |
| C-type lectin domain family 4 member E | Clec4e | 21.715 | 16 | 3 | 24.4 |
| Platelet factor 4 | Pf4 | 9.181 | 29 | 3 | 11.2 |
| C-X-C motif chemokine 10 | Cxcl10 | 9.972 | 24 | 2 | 10.8 |
| Pentraxin-related protein PTX3 | Ptx3 | 28.399 | 17 | 6 | 41.8 |
| Stromelysin-1 | Mmp3 | 26.803 | 16 | 7 | 53.8 |
| Isoform 2 of Sequestosome-1 | Sqstm1 | 82.623 | 38 | 10 | 44.2 |
| Neutrophil gelatinase-associated lipocalin | Lcn2 | 13.712 | 18 | 3 | 22.9 |
| CD82 antigen | Cd82 | 16.885 | 13 | 3 | 29.6 |
| Tumor necrosis factor receptor superfamily member 1B | Tnfrsf1b | 17.745 | 10 | 3 | 50.3 |
| Copper transport protein ATOX1 | Atox1 | 8.973 | 38 | 2 | 7.3 |
| Prostaglandin G/H synthase 2 | Ptgs2 | 12.982 | 8 | 4 | 69 |
| Intercellular adhesion molecule 1 | Icam1 | 28.385 | 13 | 7 | 58.8 |
| GRIP1-associated protein 1 | Gripap1 | 13.922 | 6 | 5 | 92.7 |
| MAGUK p55 subfamily member 6 | Mpp6 | 4.646 | 4 | 2 | 62.6 |
